# Knowledge, Attitude and Practice of mothers on acute respiratory infection in children under five years

**DOI:** 10.12669/pjms.326.10788

**Published:** 2016

**Authors:** Shireen Qassim Bham, Farhan Saeed, Manzar Alam Shah

**Affiliations:** 1 Dr. Shireen Qasim Bham, DCH, FCPS. Department of Pediatrics, Liaquat College of Medicine & Dentistry and Darul Sehat Hospital, Karachi, Pakistan; 2 Dr. Farhan Saeed, DCH, FCPS. Department of Pediatrics, Liaquat College of Medicine & Dentistry and Darul Sehat Hospital, Karachi, Pakistan; 3 Dr. Manzar Alam Shah, MPH. Department of Community Medicine, Liaquat College of Medicine & Dentistry and Darul Sehat Hospital, Karachi, Pakistan

**Keywords:** Acute Respiratory Infection(ARI), Expanded program of Immunization(EPI), KAP mother on ARI. Pneumonia in children

## Abstract

**Objective:**

To assess Knowledge, Attitude and Practices of mothers on ARI (Acute Respiratory Tract Infection) in children less than five years of age.

**Methods:**

This cross-sectional survey was conducted in the Department of Pediatrics, Darul Sehat hospital from 1^st^ December 2014 to 28^th^ February 2015. Mothers(n=335) who were local residents, had at least one child below the age of five years and coming to the hospital for any medical problem along with accompanying women were included. Foreign mothers and/or those having difficulty in perceiving questions were excluded. Language used in the Questionnaire was English which was translated to Urdu for better understanding. Questionnaire was interviewer administered. Researchers and two house physicians took part in questioning the mothers.

**Results:**

Total 335 children were studied. Out of 335 children 228(68%) had ARI. Mean age of the children was 20 months ±17 SD while mean Birth weight was 2.7 kg ± 1.8 SD. The most common symptom perceived was cough (n=303, 40%), mostly worsening during winter season (n=255,87%), commonest aggravating factor was dust (n=174,81%), most common complication was Pneumonia (n=135, 83%), and most mothers opted for medical practitioner (n=268,89%) for treatment. Self-medication was practiced by 192(58%) and paracetamol was frequently used medication (n=117,42%).

**Conclusion:**

The study reveals good knowledge of mothers on ARI symptoms, worsening environmental conditions, aggravating factors and complications. Their attitude towards ARI was appropriate with early consultation with qualified medical practitioner. Better literacy rate, has a positive influence on the Knowledge, Attitude and Practices of mothers.

## INTRODUCTION

Acute Respiratory Infection (ARI) is considered as one of the leading causes of morbidity and mortality in children and it incurs upon high economic cost. It is the main reason for utilization of health services for children. Its control is a big public health concern especially in developing countries. It constitutes Upper Respiratory Infection (URI) and Lower Respiratory Infection (LRI). Upper respiratory infection presents mainly with Rhinitis (Common Cold), Tonsillitis, Sinusitis and ear infection while main presentations of LRI is Pneumonia which exhibits with increased respiratory rate

All over the world, average ARI experience of child is 6-8 spells in a year.^1^ The incidence of ARI in Pakistan is 16% as shown by a survey conducted in Pakistan in 2011.^2^ That survey also revealed that ARI was more prevalent in urban areas in the country. Over the counter (OTC) drugs are frequently administered by parents to their children as ARI causes discomfort and distress to the parents. Proven efficacy of such drugs is lacking.^3^ Also these could be hazardous^4^ and have not been endorsed by Food and Drug Administration (FDA)^5^ and American Academy of Pediatrics.^6^ In such conditions safe home remedy and proper care are mainly recommended.

In developing countries, awareness regarding knowledge attitude and practices of mothers in acute respiratory tract infection (ARI) needed evaluation so researchers endeavored to get base line data for better understanding of the magnitude of problem. That will add to the existing pool of knowledge on ARI. The objective of the study was to assess knowledge, attitude and practices of mother’s on ARI(Acute Respiratory Tract Infection) in children less than five years.

## METHODS

This cross-sectional survey was conducted in the Department of Pediatrics, Darul Sehat hospital, Karachi from December 01, 2014 till February 28, 2015 as there is more reported cases of ARI during winter season in our hospital. It was non probability purposive sampling. Keeping incidence of ARI 16%, CI 95% and absolute precision required 0.05, minimum sample size came out 204.

### Inclusion criteria

Mothers who opted to participate in the study. Mothers who had at least one child below the age of five years and who came to the hospital for any of their child’s disorder. Mothers coming to the hospital for any of their medical problem. Women who were accompanying them and had at least one child less than five years of age

### Exclusion criteria

Mothers unable to perceive questions. Mothers who are foreigner to the local area

Permission was taken from the Ethical Review Committee. Study protocol was approved by Ethical Review Committee of Liaquat College of Medicine & Dentistry on 10^th^ November 2014. Verbal informed consent was taken from the mothers. Their autonomy, confidentiality and anonymity were maintained. They had complete liberty not to response to any of the questions and quit at any time during the study. That act would, in no way, affect their due care in the hospital.

### Data collection procedure

Language used in the Questionnaire was English which was translated to Urdu for better understanding. Questionnaires were interviewer administered. Researchers and two house physicians took part in questioning the mothers. The researchers scrutinized these filled questionnaires. Main Independent variables included gender, birth weight and mothers education. The main dependent variables were knowledge of mothers on symptoms of ARI, disease aggravating and worsening factors and complications of the disease. Education, occupation, parity of the mothers, socioeconomic status and family type were categorical variables. Practicing self- medication, type of self- medication and consulting qualified persons for the disease were also dependent variables.

### Data analysis

SPSS-16 was used. For birth weight mean and standard deviation while for other independent and categorical variables frequencies were calculated. Missing data on child birth weight was 18% and was replaced with series mean.

## RESULTS

Total 335 mothers were interviewed. Out of 335 children 228(68%) had ARI. Two hundred sixty five (81%) of mothers acquired higher than secondary education (Table-I). Two hundred ninety six (92%) mothers were house wives and 216 (66%) mothers had less than two children. Mean age of the children was 20 months ±17 SD while that of the mothers was 29 years ± 4 SD. Mean birth weight of the children was 2.7 kgs ±1.8 SD. Mean duration of ARI was five days (SD2.1). Two hundred twenty (85%) had monthly earning of > Rs.20, 000/m. Joint family system constituted 201 (62%) and 325 (99%) of the children were delivered at hospital. Fully vaccinated children by EPI were 309 (94%) while 261 (80%) were vaccinated against Pneumonia (Table-I). Only 36 (11%) of the children were suffering from under nutrition and 229 (69%) were breast fed (Table-I).

**Table-I:** Baseline Socio-Demographic Characteristics of Study Population (n=335)

Mothers Characteristics	Frequency	Percentage
*Education (n=328)*		
No education	10	3
Non formal education	4	1
Primary	5	2
Secondary	11	3
Matriculate	33	10
Higher	265	81
*Occupation (n=323)*		
House wife	297	92
Working women	26	8
*Number of children (n=329)*		
< 2 children	216	66
3-4 children 106	32	
5 or more children	7	2
*Monthly household income per month (n=262)*		
< Rs. 10,000	6	2
Rs. 10,000 to Rs.20,000	36	13
> Rs. 20,000	220	85
*Type of family (n=326)*		
Joint	201	62
Nuclear	125	38
***Children Characteristics***		
*Gender (n=335)*		
Male	172	51
Female	163	49
*Place of delivery (n=330)*		
Hospital	325	99
Home	5	1
*Birth attended by qualified person (n=329)*		
Yes	322	98
No	7	2
*Term of the child (n=328)*		
Full term	304	93
Pre term	24	7
*Fully vaccinated children by EPI (n=329)*		
Yes	309	94
No	20	6
*Number of malnourished children (n=314)*		
No	278	89
Yes	36	11
*Number of breast fed children (n=328)*		
Yes	225	69
No	103	31

The most symptom perceived was cough (n=303, 40%), most common worsening environment was winter (n=255, 87%), most common aggravating factor was dust (n=174,81%), most common complication was Pneumonia (n=135, 83%), and most common treatment option was through medical practitioner (n=268,89%, Table-II). Self medication was practiced by (n=192, 58%) and in that use of Paracetamol was most frequent (n=117, 42%, Table-II).

**Table-II:** Knowledge, Attitude and practice of mothers on ARI (n=335).

	Frequency	Percent
***Knowledge***		
*What are the symptoms of ARI?*		
Cough	303	40
Fever	255	34
Wheezing	67	9
Sneezing	88	12
Pain in ear, nose, throat	41	5
*Disease worsening environmental condition*		
Summer	21	7
Winter	255	87
Autumn	13	4
Rain	5	2
*Aggravating factors of the disease*		
Dust	174	81
Over crowding	29	13
Poverty	6	3
No immunization	7	3
*Complications of ARI*		
Fits	14	9
Pneumonia	135	83
Ear discharge	10	6
Measles	4	2
*Treatment options for ARI*		
Consulted qualified doctor	268	89
Did not consulted doctor	17	6
Bed rest	9	3
Home remedy	19	6
Don’t know	6	2
***Practice***		
*Practicing self medication in ARI*		
Yes	192	58
No	142	42
*Type of self medication*		
Ibuprofen	61	22
Paracetamol	117	42
Anti allergy	64	23
Anti biotics	21	8
Joshanda	11	4
Homeopathy	5	1
Honey	1	0.5

## DISCUSSION

Mothers were interviewed in this study because mostly mothers accompany their children to hospital.^7^,^8^ Most of the mothers had less than two children. The study showed an overall literacy rate of mothers being 97%. Of these, 80% were higher than secondary education. This is in comparison to a similar study^9^ conducted at Tharparkar showing overall literacy rate of mothers being 74% with urban background. Both the studies are showing higher level of education in mothers as they are having urban background and this fact lays emphasis that the government should make efforts to increase the level of education in mothers in rural areas. Literate mothers are more vigilant in seeking medical care for their children.

Immunization coverage of children by EPI in this study was 94% where as it is 85% as shown by a study conducted in Kenya.^10^ This study showed that 69% of the children with ARI were continued breast feeding while in another study it was 65%.^11^

In this study 36 (11%) of the children was suffering from under nutrition while in a study conducted in Nepal^12^ it was 23 (38%). This finding stresses upon the concerned authorities to start health programs regarding nutrition of children. Such programs are likely to be successful as literacy rate of mothers is high and they mostly belong to working class.

The most common symptom of ARI found in this study was Cough (40%). Other symptoms in order of frequency were Fever (34%), Wheeze (9%), Sneezing (12%) and ear ache (5%) where as in a study carried out in Ghana the common symptoms were retraction of ribs (22%), fever cough and lethargy (57%)^13^ In a study conducted in Dar us Salam major symptoms were Fever (92.5%), Cough (85.3%) and inability to play (83.5%).^14^

The mean duration of ARI in this study was five days (SD2.1) which is in contrast to a study conducted by Shahzad Munir^11^ showing mean of 4.5 days (SD-3.1). This low mean duration of the illness could be due to the fact that 317 (94%) mothers consulted qualified medical practitioner for ARI where as in a study conducted in Tehran^15^, 141 (62%) mothers preferred medical practitioner.

In this study 8% of the mothers believed that antibiotics were necessary for ARI. In other studies higher incidence of use of antibiotic has been reported by Chan *et al*.^16^ (68%) and Bhanwra *et al*.^17^ (46%). These are contradictory to our findings as mothers are more literate having better awareness about the hazards of using unnecessary antibiotics. Studies conducted by Farhad *et al*.^18^ showed use of antibiotics 5% and Panagakau *et al.^19^* 10%. These percentages are similar to study in hand. Less use of antibiotic in this study is a healthy sign showing that mothers are well aware of this important issue.

Use of self medication was found 58% in this study. Similar picture was shown by a study conducted in Multan^20^ where it was 58%. The self medication used in this study includes paracetamol and ibuprofen which is extremely common and the mothers think that such drugs are harmless and could safely be given. Such drugs are over-the-counter class of drugs and that routinely used as self-medication reason being less harm full.

Home remedies were practiced by 6% of the participants in the current study. Other studies conducted in, Multan^20^ and Lahore^21^ demonstrate practice of home remedies as 40% and 23% respectively. Joshanda use was 4% of the self medication. In a study conducted in New Delhi^22^ the use of Ginger as home remedy in ARI in children was 27%. This could be due to cultural difference.

Dust was the most common aggravating factor of the disease (81%) while in a study conducted in Myanmar it was 89%.^23^ This finding could be due to the general environmental condition of area and low municipality services.

## CONCLUSION

Knowledge of mothers on ARI symptoms, worsening environmental conditions, aggravating factors and complications was found satisfactory. Their attitude towards ARI was appropriate with early consultation with qualified medical practitioner. Better literacy rate, has a positive influence on the Knowledge, Attitude and Practices of mothers.
